# On the Medical Effects of Phosphorus

**Published:** 1803-04-01

**Authors:** 


### *On the* Medical Effects *of* Phosphorus; *communicated by our Correspondent at Paris.*

FROM accounts which were published some time back in London, on the medical use of phosphorus, it appeared that this substance held a distinguished rank among the class of alexipharmics, and that it had been employed in a variety of cases with success, in restoring the vital energies. The Medical Society of Paris proposed, in the sixth year, the following question. "To determine, by experiment and observation, what are the medical properties of phosphorus, of the phosphoric acids, in what cases they may be employed with success, and what is their mode of action." The memoirs which were sent in consequence, were not conceived to answer the object, and the question was adjourned.

In the year 7, Cit. Sedillot published an account in one of the Journals of some observations which tend to the support of those already made in London, as well as of a memoir by Dr. Lentin of Gottingen, *De acido phosphori cariei ossium domitore;* in which the author proves phosphorus to be one of the best remedies with which the materia medica can be enriched. M. C. Pelletier pointed out in the Recueil Periodique, vol. 9, the best mode of keeping phosphorus deposited in sulphuric æther, so as to be able to regulate the dose; since when, a number of French physicians have obtained results from its use, which deserve notice, but which, owing to a wise precaution of the commission named to observe these effects, have not as yet been been made public. In the mean time we think it useful to publish such isolated facts as come to our knowledge, which may help to place this medicine in its proper place, and the following seems calculated to answer this end.

*On the Effects of phosphorated Æther in a Case of Paralysis and Atony of the Fibre, accompanied by Infiltration of Water in the cellular Membrane; by* M. Gaultier Chaubry; *read at the Medical Society,* 12 *Germinal, Year* 10.

OBSERVATION 1. Jean D. aged 40 years, fell sick on the 5th Brumaire, (27 Oct.). He was carried to the Hotel Dieu, labouring under putrid fever, for which *he was bled five times.* His legs and thighs were swoln, and he was extremely weak. A strong desire to revisit his native city, Rouen, induced him to quit the hospital the 24 Frimaire, (15 Dec.). The 25th, in the morning, the swelling had manifested itself in the scrotum and penis, and but little urine flowed. On the 26th the belly became distended, and the patient complained of a sensation as if girted by a large band. The pulse was small and slow. I conceived, says the author, this to be a fair opportunity for exhibiting the phosphorated ether, which I did to the quantity of ten drops in a common vehicle. The patient made a considerable quantity of water during the night. On the morning of the 27th the tension of the belly and the swelling of the scrotum and penis had diminished. I increased this day the dose from ten to fifteen drops, to be taken in three portions, at the interval of three hours. The urine flowed abundantly. 29th. The same quantity was administered with the same results and the addition of two stools. The 30th the swelling had disappeared from the scrotum, belly, and the greater part of the thighs. 1st and 2d Nivose, (Dec. 23,) the same treatment was continued and no swelling remained. His strength and appetite began to return; I gave more nourishment and eight drops of ether, which he continued in the same quantities from the 5th to the 8th. The 9th he experienced an appetite for food as well as for other enjoyments, and I entirely discontinued the medicine. The author concludes by observing that 117 drops of the phosphorated ether cured this patient, in which quantity he computes about 1 [illegible]/5 grains of phosphorus.

In a second observation, the author tells us of a woman, whose menstrual discharges disappeared at the usual period, without any inconvenience; she was now fifty-five years of age, had suckled five children, and came to Paris on on business, where she was attacked the 30th of Vendemaire, (Sept. 23,) with putrid fever, which continued till the 27th of November, after which her legs and thighs swelled, and she had hardly the power of motion; her inferior extremities were generally cold. Urine came, but in small quantities, and the pulse was hardly perceptible. The phosphorated ether was administered as in the preceding case; the following night the patient passed somewhat more water, but the weakness was excessive, the pale colour of the face frightful, &c. The morning following the appearances were nearly the same, except that the extremities were become completely cold; ten drops of ether were given at three o'clock, and the same dose was repeated at nine. The pulse was become fuller and somewhat more appearance of heat in the lower extremities. 30th. At four o'clock she made a good deal of water; the pulse was weaker; the heat of the body was somewhat increased; the extremities still remained cold. Her legs and thighs were rubbed with phosphorated ether, and she took internally ten drops. At twelve o'clock the patient had stools and passed water. The heat and motion in the extremities, suspended for twenty days, began to return.

The 1st Nivose (Dec. 22,) I employed frictions in the morning, and six drops of ether three times a day; the power of motion continued to increase, and the swelling of the legs began to disappear. The 2d, 3d, 4th, and 5th, ten drops of ether were administered. On the 6th, the patient found herself well; a suitable diet was recommended, together with frictions with flannel. She had taken 128 drops of the ether independent of what had been used in the form of frictions.

The author gives some other cases, nearly similar in their nature, where he used with the same advantage the phosphorated ether; in one of them, where the stomach loathed the phosphorus, it was found that the addition of a few drops of ætheris acetati made it more palatable.

The author means to give, on a future occasion, an account of the successful use of this medicine in other cases, and concludes his paper by giving directions with respect to its administration. He recommends us to ascertain with the greatest possible accuracy, whether or not the patient labours under any particular irritation, or if inflammation be the cause of those complaints which we have to combat. He thinks it necessary to keep the ether in a bottle with a glass stopple, and to agitate the materials in it; he has not perceived that the phosphorus separated from the liquor with with which it was combined, he only remarked that when exposed to much heat, or to the rays of the sun, the liquor became turbid; to avoid these inconveniences it is necessary to keep the liquor in a cool and dark place, and to wrap the phial in a piece of black cloth.

